# Transforming patient management: a study on secure, cost-effective, automated remote monitoring of urine bags

**DOI:** 10.1038/s41598-025-24141-1

**Published:** 2025-11-17

**Authors:** Anil Kumar Bhat, G. R. Pradyumna, K. B. Bommegowda, Roopa B. Hegde, Swathi Prabhu

**Affiliations:** 1 Department of Electronics & Communication Engineering, Nitte (Deemed to be University), NMAM Institute of Technology (NMAMIT), Nitte, 574110 Karnataka India; 2 Manipal Institute of Technology, Manipal Academy of Higher Education, Manipal, 576104 Karnataka India

**Keywords:** Patient monitoring, Urine output, Capacitive sensors, Remote monitoring, Wireless transmission, Health care, Medical research, Electrical and electronic engineering

## Abstract

The increasing demand for efficient patient monitoring systems in healthcare and the growing need for remote monitoring, particularly post-pandemic, emphasise the importance of tracking critical parameters such as urine output, blood oxygen saturation, breath rate, and blood pressure. Urine output, a key indicator of kidney function and medical treatment response, is traditionally assessed manually, posing a significant burden on hospital staff and caregivers. Addressing this, our system facilitates continuous, accurate monitoring of urine output, enhancing patient care and healthcare efficiency. We developed a smartphone application leveraging capacitive sensors and a Wi-Fi-enabled control unit, enabling remote monitoring of urine bag volumes. The system alerts when bags are empty for extended periods or full, this is validated through experiments with volumes ranging from 100 to 1000 mL.The corresponding variations in sensor output voltage confirmed the accuracy of the system. To secure patient data, we incorporated AES-256 encryption with dynamic key generation using patient-specific IDs and OTP-based access control, ensuring data privacy and compliance with healthcare regulations. Our approach offers several advantages: ease of attachment to standard urine bags, non-invasiveness, reusability of bags, and remote monitoring through the mobile application. This innovation automates urine output monitoring, secures patient data, reduces the workload of intensive care nurses, and enhances patient care through precise and continuous monitoring. Unlike existing devices that rely on customised containers or short-range Bluetooth transmission, our system is compatible with standard urine bags, employs cost-effective capacitive copper-tape sensors, and integrates AES-256 encryption with dynamic key generation and OTP-based access control for robust data security. These unique features make the system functionally novel, technically secure, and highly practical for deployment in both hospital and home settings.

## Introduction

Patient monitoring involves continuously observing physiological functions to enhance patient outcomes. This continuous monitoring is crucial in-home care and remote consultations. Physicians rely on real-time physiological data from healthcare staff or home caregivers to make critical decisions. A study^1^ highlighted the rising ICU admission rates, which increased the demand for additional personnel and increased strain on the healthcare system. However, a survey by Poncette et al.^2^ reveals that hospital staff and nurses strongly desire automation in patient monitoring systems. Monitoring devices can measure physiological functions and parameters as blood oxygen saturation and breath rate. These devices display parameter values and issue audible alerts if values deviate from the normal range^3^. In situations where accurate decision-making is essential for effective patient care, electronic monitors collect and display physiological data. Non-invasive sensors, used in medical centres or patients’ homes, help detect unexpected life-threatening situations or efficiently record routine data. As a result, remote monitoring eases the workload of hospital staff. Introducing automation and remote monitoring facilities significantly enhances patient care, reducing mortality rates due to inadequate attention or continuous monitoring.

Monitoring Urine Output (UO) is carried out manually and is a crucial indicator of a patient’s kidney health^4^. Sufficient urine reflects well-perfused and oxygenated kidneys in hospitals or home care settings. The typical range for a 24 h urine volume is 800 to 2000 mL, with an average daily fluid intake of about two litres. Ideally, a person should pass at least 100 mL of urine every 6 h; otherwise, it may indicate a pathological disorder^3^. UO not only helps diagnose conditions like anuria (no UO), oliguria (low UO), or polyuria (high UO) based on volume but also aids physicians in evaluating a patient’s response to medical treatment and calculating the body’s water balance. Currently, in critically ill patients, UO is manually measured at intervals for clinical decisions. However, transitioning to minute–to–minute monitoring with an automatic device could save valuable time for healthcare staff and significantly enhance patient care by providing more precise and continuous monitoring of this vital parameter.

The healthcare sector faces many problems, including insufficient resources, unaffordable systems, and inefficient management of hospital resources^5^. Regular patient monitoring can reduce the risk of severe issues and facilitate timely treatment. Traditional manual monitoring burdens the supporting staff, and non-availability may lead to serious problems. The digital era and the Internet of Things (IoT) have opened new opportunities for improving the delivery of healthcare services, with remote monitoring systems playing a crucial role in the medical sector^6^. The application of existing technologies for automated UO monitoring has significant benefits, such as simplifying the task and increasing the monitoring efficiency of patients.

Several studies have been carried out in recent years on human health monitoring systems based on IoT. This use of technology in the healthcare sector for remote monitoring of patients has gained popularity during the pandemic. Significant progress can be witnessed in remote consultation and remote monitoring of various medical data. This technological advancement in the healthcare system has contributed to safety, health, and human well-being. Research in automated health monitoring systems using IoT is geared up to advance the healthcare system. Integration of several sensor systems for monitoring day–to–day activities of health constraints and taking necessary action in timely events^7^ is widespread nowadays. Several remote monitoring systems to monitor blood pressure, heart rate, oxygen level, temperature, breath rate, and other daily activities have been found in recent years^8–12^. Das et al.^13^ disclosed that innovative biosensors could record and detect human physiological characteristics. However, it is also necessary to design a system that can monitor patients whenever they are moving. The General Packet Radio Services (GPRS) technology allows real-time monitoring of moving patients’ heart rates and temperatures^14^. A recent development using the Global System for Mobile Communication (GSM) module made it caution doctors through Short Message Service (SMS) in case of any variation in a patient’s heartbeat and Blood Pressure (BP)^15^. Nowadays, monitoring older adults and providing quick aid in case of falls is essential. This led to the design of mobile biodevices for monitoring the outdoor activities of the aged population^16^, and it was concluded that thousands of older adults could be monitored simultaneously. The development of IoT-based remote monitoring led to the investigation of the feasibility of remote UO-level monitoring.

A study on electronic UO monitoring versus manual monitoring in 2021^17^ reported that electronic UO-level monitoring is more accurate than manual monitoring. Another investigation found that an electronic UO monitor provides stable readings from time to time and could be used for early detection of Acute Kidney Injury (AKI) by incorporating automated analysis of urine output^18^. However, much earlier in this study, automated UO level monitoring is proposed to provide minute-by-minute UO, with the device capable of emptying the bag when it is full^2^. The system requires an extra container to empty the bag. Bluetooth technology is employed for wireless monitoring of UO^19^ to eliminate the wired transmission. Remote monitoring is achieved in a limited range with Bluetooth transmission. Recently, an automated weight based UO level monitoring system has been developed^20^ for monitoring ICU patients. The system could be used by tying the bag to the patient’s bed. An analysis of the design and optimization of real-time UO level explored that using IoT in automated monitoring of UO can make the system accurate and efficient^21^, thereby strengthening the nursing workflow. Advancements in IoT and wireless communication technology led many research groups to design an automated system for UO monitoring either for hospital use or home care^22,23^. Additionally, various UO-level monitoring systems have been proposed by employing a range of sensors, including capacitive sensors^24^, weight sensors^25^, float sensors^26^, and coaxial capacitive sensors^27^. However, capacitive sensors are integrated into rigid containers, and flow sensors are combined with Foley catheters. These studies assure possibilities for designing real-time UO-level monitoring systems, and there is a requirement for such systems in healthcare units. Comprehensive research revealed the state-of-the-art methods of IoT and sensor-based devices for incontinence and automatic monitoring of UO. Such devices enable early detection of potential health issues, such as kidney dysfunction or urinary tract problems, by promptly identifying changes in UO patterns. Also, such systems can address the need for continuous, accurate, and timely monitoring of patients’ urine output, leading to improved patient care, early detection of health issues, and efficient healthcare delivery.

While several electronic urine output monitoring systems have been reported^3,17-27^, they often suffer from limitations such as dependence on customised urine containers^3,24,26^, short-range Bluetooth transmission without true remote access^19^, or lack of integrated data security^25–27^. In contrast, the proposed system is functionally and technically distinct in several ways: (a) Compatibility with standard urine bags, avoiding the need for specialised or invasive containers, (b) Use of low-cost copper tape-based capacitive sensors, reducing the overall system cost to approximately 15 USD, making it far more affordable than most reported solutions, (c) Integration of AES-256 encryption with dynamic key generation and OTP-based access control, addressing a critical gap in secure patient data transmission for remote monitoring applications, (d) Deployment of a Wi-Fi-enabled control unit with smartphone app support, which enables real-time remote monitoring without the range limitations of Bluetooth-based systems. These features collectively establish the technical novelty of our approach, positioning it as a secure, low-cost, and practically deployable solution for automated urine output monitoring.

The proposed system for monitoring UO levels is based on an Android application (App) designed to monitor urine levels remotely. This system ensures continuous monitoring of UO levels and sends real-time updates to the concerned individual’s mobile phone via the App. It utilizes a non-invasive approach by implementing a capacitive sensor to track urine levels. The key contributions of this work are as follows.

1. Development of a non-invasive urine level monitoring system compatible with commonly available urine bags.
2. Development of time and cost-effective remote monitoring system using Wi-Fi technology.
3. Development of a user-friendly cell phone App that enables remote monitoring of urine levels.
4. Implement an alerting system that triggers alarms in the event of no urination within a 6-hour.
5. To develop a secure and efficient remote urine output monitoring system using AES-256 encryption with dynamic keys and OTP-based access control, ensuring patient data confidentiality and integrity for enhanced healthcare delivery.

This system aims to enhance healthcare monitoring by providing a convenient and cost-effective solution for tracking UO levels and ensuring timely alerts when necessary. The significant contribution of the proposed work is the non-invasive approach of UO level measurement with the existing urine bag. The novelty lies in the implementation of the proposed system with standard urine bags that are available in the market. Additionally, remote monitoring using a smartphone App and integration of AES-256 encryption and dynamic key generation for secure data transmission, addressing the critical aspect of data privacy and security.

## Development of an automated level monitoring system

Design and development details corresponding to hardware and software are provided in this section. An overview of the system and details of the device development and working principle are provided below.

### Overview of the non-invasive UO level indicator system

**Fig. 1:**
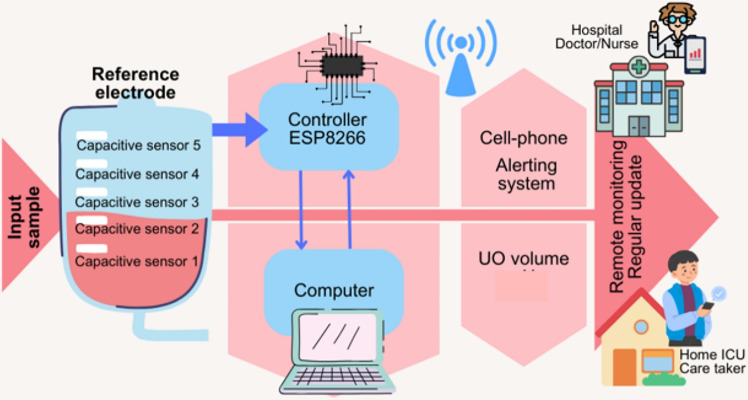
The working scenario of the proposed UO-level monitoring.

The proposed non-invasive urine bag monitoring system is illustrated in Fig. 1. Capacitive sensors are strategically positioned on the exterior of the urine bag to detect various UO levels and relay this vital information to a central control unit. This control unit is connected to the sensors on the bag via a wired connection, and either batteries or an AC mains source can power it.

The ESP8266 controller board continuously monitors the output voltage values generated by the sensors. These voltage readings are then transmitted wirelessly to a designated cell phone, where they are accessible through a dedicated Android App. A mobile phone can monitor multiple such systems by installing the App. Systems. The system supports multiple authorised mobile devices accessing data from the same urine bag simultaneously. This is enabled through the Wi-Fi module and server-based data synchronisation, allowing doctors and nurses to monitor a patient in parallel. A single mobile phone can monitor multiple such systems by installing the App. Systems. This wireless communication facilitates real-time monitoring and timely response. We integrated advanced encryption methods to enhance patient data security, utilizing AES-256 encryption coupled with dynamic key generation based on patient-specific IDs and an OTP-based access control system. These measures ensure the secure transmission and access of sensitive data, addressing concerns related to data privacy and compliance with healthcare regulations. To ensure patient safety, the system has an initialization sequence for the sensors, transducer, and timer circuits. If, even after 6 hours, the urine level remains below 10%, the system automatically generates an alert message, notifying the relevant individual to take immediate action. Also, an alert message is sent if the urine bag is full. This comprehensive system provides accurate and continuous urine bag monitoring, enhancing patient care and ensuring timely interventions when necessary.

**Fig. 2:**
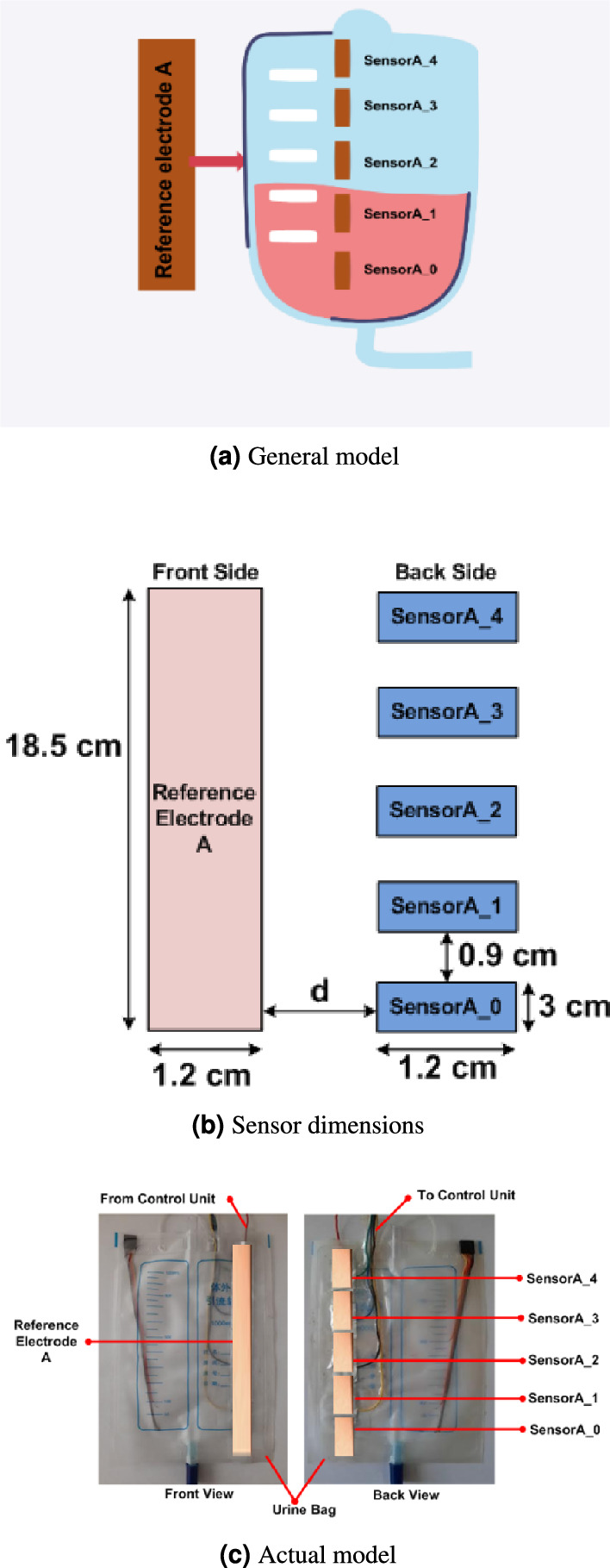
Alignment of sensors on urine bag.

Capacitive sensors offer a contactless method for detecting both solid and liquid targets. They operate by sensing changes in the electrical field across a charged capacitor which leads to the presence of a target. Essentially, capacitive sensors function similarly to regular capacitors. In the proposed system, we employ copper tape with a length of 18.5 cm and a width of 1.2 cm, adhered to the front surface of the urine bag. This serves as the reference plate for the sensor electrode. The control unit generates a 100 kHz signal applied to the reference electrode. The sensor electrode comprises five metal electrodes (labelled SensorA_0 to SensorA_4), each measuring 3 cm in length and 1.2 cm in width, with a gap of 0.9 cm between the sensors, as shown in Fig. 2b. These electrodes are fixed to the back surface of the bag, precisely opposite to the reference electrode, forming a broad-side coupled capacitor configuration. The sensor arrangements are shown in Fig. 2. This arrangement enables effective detection and monitoring of urine levels within the bag, contributing to the system’s functionality. Experimentations were conducted with normal and saline water to test the proposed UO monitoring system. In the experimentation, we used medical-grade normal saline water with a pH of 5.5 and salinity of 0.9%. This equates to 9 grams of salt per litre of water.

### Working principle of the automated UO monitoring system

The system works based on the capacitive principle. The sensor electrode fixed to the medical-grade polyvinyl chloride (PVC) urine bag can be represented as a parallel plate capacitor. During the operation, the value of the capacitance of the sensor changes due to changes in dielectric material as well as the distance between the electrode plates. The changes in the capacitance, on the application of 100 kHz square wave, results in a voltage variation detected by the analogue-to-digital converter (ADC) integrated into the control unit.

#### Circuit level analysis

**Fig. 3:**
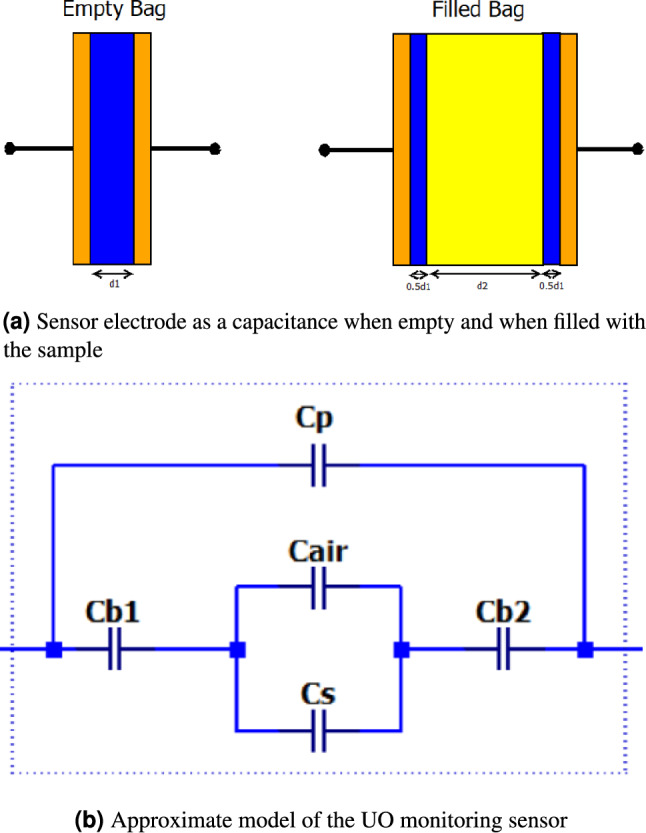
Sensor capacitance model.

The sensor capacitance can be represented as a series-parallel combination of capacitors, as illustrated in Fig. 3, and here, dimension d1 corresponds to the thickness of the urine bag (0.1 mm). In contrast, dimension d2 corresponds to the varying gap created by the sample. Within this illustration, Cb1 & Cb2 denote the capacitance resulting from the urine bag’s material, which remains constant due to consistent electrode dimensions and bag thickness. On the other hand, Cs and Ca represent the variable capacitance due to the presence of the sample and air, respectively. Additionally, Cp accounts for the parasitic capacitance resulting from fringing effects. This capacitive model enables quantifying changes in the urine bag’s capacitance as it is used.

**Fig. 4:**
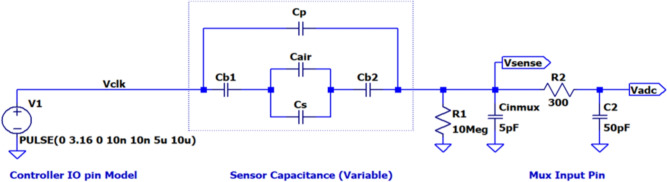
Approximate equivalent circuit model of the experimental setup.

Further, values of the capacitances are calculated using Eqs. (1) to (4). In the following equations, L represents the length of the sensor [3 cm], W the width of the sensor [1.2 cm], $\varepsilon _{o}$ represents the permittivity, $\varepsilon _{rb}$, $\varepsilon _{rs}$, and $\varepsilon _{ra}$ represent the dielectric constants of the bag, sample, and air, respectively. $L_{a}$ and $L_{s}$ correspond to the length occupied by air and sample within the electrode.

1 $$\begin{aligned} C_{b1} = C_{b2}&= \frac{2 \varepsilon _0 \varepsilon _{rb} LW}{d_1} \quad (\text {pF}), \end{aligned}$$

2 $$\begin{aligned} C_a&= \frac{\varepsilon _0 \varepsilon _{ra} L_{a}W}{d_2} \quad (\text {pF}), \end{aligned}$$

3 $$\begin{aligned} C_s&= \frac{\varepsilon _0 \varepsilon _{rs} L_s W}{d_2} \quad (\text {pF}), \end{aligned}$$

4 $$\begin{aligned} C_p&= \frac{\varepsilon _0 \varepsilon _{rb} \Delta f (L + W + \Delta f)}{d_1 + d_2} \quad (\text {pF}). \end{aligned}$$

The value of the fringing coefficient $\Delta {f}$, is computed using equation (5). The thickness of the bag and the thickness between the plates are represented by *d*1 and *d*2, respectively.

5 $$\begin{aligned} \Delta f&= d_1 + d_2 + \frac{0.0885 (d_1 + d_2) \ln (LW + 1)}{\pi } \quad (\text {cm}). \end{aligned}$$

During the operation, the possible variations are summarised under three conditions.

- Condition-1: The bag is dry and empty, essentially a new, unused bag. In this case, the sample is not present. Hence Cs component does not exist. Electrode capacitance is mainly due to the bag capacitance Cb in series with Ca and parallel with the parasitic capacitance Cp.
- Condition-2: A small sample is present in the bag, so the sample level does not entirely cover the sensor electrode. In this case, both Ca and Cs are nonzero quantities. Since they are parallel, their capacitance adds up, which is in series with Cb. Hence, the effective electrode capacitance value is higher than in Condition-1. Initially, when a test sample is added to the bag, there is a sudden increase in the value of the Cs. This is because the dielectric constant of the sample is higher than that of the air. The capacitance decreases on further addition of the test sample as the distance between the plates increases.
- Condition-3: When the sample volume is large enough to cover the entire length of the sensor electrode, Ca = 0. As the volume of the sample increases, the distance between the plates increases, resulting in a decrease in the value of the Cs. As the volume increases, the bag bulges and Cp increases due to bulging the bag. So, the reduction in CS is, to a certain extent, compensated by the increase in Cp

To test the correctness of the model, simulation is carried out using the LTspice$\circledR$ simulation tool. The entire measurement setup is modelled as shown in Fig. 4, where $C_b$, $C_a$ and $C_s$ are the bags, air, and sample capacitance, respectively. Cinmux represents the input capacitance of the analog multiplexer and serves as a load for the measurement system. Component $R_2$ is the resistance of the Multiplexer, and $C_2$ is its internal capacitance that connects to the circuit when the multiplexer input is selected. The average ADC reading is estimated using the ‘.meas’ command.

The various parameter values considered for simulation and the computed capacitance values are tabulated in Table 1. Also, the average output voltage values obtained in the simulation for the different sample volumes are listed. As observed from the table, the simulated values show a sudden increase in output voltage for the case when there is no sample and when there is 100 mL of the sample. Also, the voltage steadily increases as capacitance increases, with the increasing sample volumes for up to 500 mL. After this, since the sample completely occupies the length of the sensor electrode, any further increase in the sample results in the distance between the plates to increase. Hence, capacitance decreases, causing a decrease in output voltage. Similar behaviour is observed in the experimental measurements shown in Fig. 8 in the results section. As the simulated model voltages closely follow the measured values, we can conclude that the model matches the system behaviour and can aid UO level estimation.

**Table 1:** Capacitance computation parameters and simulation results.

Parameter	Empty	100 mL	200 mL	300 mL	500 mL	800 mL	1000 mL
Dielectric constant of urine bag (PVC) $(\varepsilon _{rb})$	3.2	–	–	–	–	–	–
Dielectric constant of the sample $(\varepsilon _{rs})$	78.4	–	–	–	–	–	–
Thickness of the urine bag (*d*1) [cm]	0.01	0.01	0.01	0.01	0.01	0.01	0.01
Thickness between the plates (*d*2) [cm]	0.001	0.375	0.5	0.65	0.7	0.8	0.825
Bag capacitance $(C_{b1}/C_{b2})$ [pF]	203.904	203.904	203.904	203.904	203.904	203.904	203.904
Air capacitance $(C_a)$ [pF]	7.965	0.567	0.213	0	0	0	0
Sample capacitance $(C_s)$ [pF]	0	22.203	33.305	38.429	35.684	31.223	30.277
Parasitic capacitance $(C_p)$ [pF]	1.733	1.926	1.999	2.084	2.114	2.172	2.185
Average voltage [mV] (simulated)	47.957	343.307	388.706	391.851	392.6	385.459	383.713

**Fig. 5:**
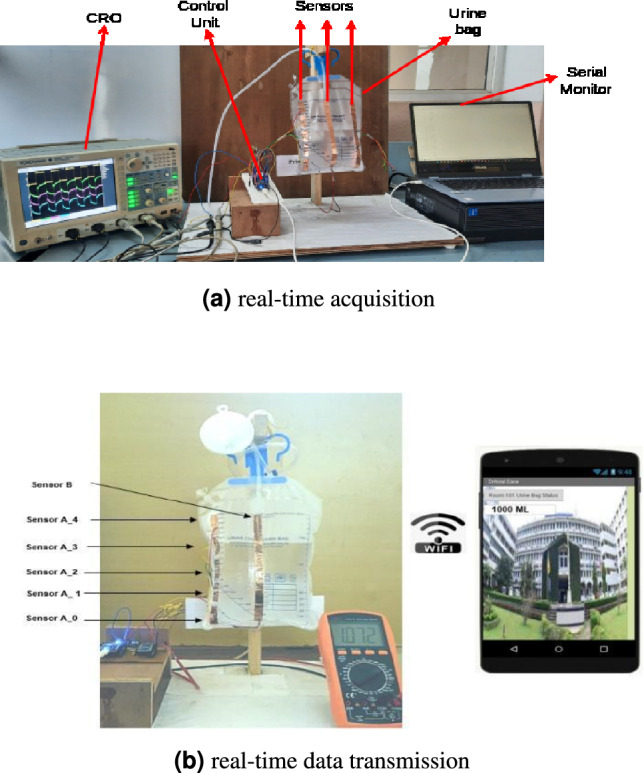
Experimental setup for UO-level monitoring.

In the present analysis, five capacitive sensors, namely Sensor A_0 to Sensor A_4, are used as depicted in the experimental setup of the remote UO monitoring in Fig. 5. These sensors are connected at different levels to monitor the different UO levels. During the experimental phase, we introduced a single stripline sensor labelled Sensor B, as depicted in Fig. 5, to assess the necessity of multiple sensors. However, the single electrode failed to provide the expected results. Hence, our analysis shifted towards considering the output of multiple sensors for estimating UO levels. As the volume within the urine bag increased, noticeable variations in the distance between the bag’s sides and the dielectric value occurred. This arrangement provided the voltage variations with varying volumes in the bag. This leads to a change in capacitance and, hence, a change in the voltage level of the sensors. Further, the variations in the voltage values are sent to the controller unit through a wired connection, as depicted in Fig. 5a . The same is sent to the mobile phone through wireless transmission, as depicted in Fig. 5b . The users can access the UO level at their convenience through the mobile App. Furthermore, an alert message is sent to the user if the UO level remains unchanged for six hours or if the bag is full.

The proposed system is enhanced with security in remote UO Monitoring. This is because in remote healthcare monitoring, the security and privacy of patient data are paramount. Our system, designed for remote UO monitoring, incorporates advanced security measures to ensure the confidentiality and integrity of patient data, addressing the critical needs of modern healthcare technology. At the core of our system is AES-256 encryption, renowned for its robustness and widely adopted in secure data environments. We enhance this encryption standard by introducing a dynamic key generation mechanism. Each patient is assigned a unique identifier, which forms a distinct encryption key for every data transmission instance when combined with a continuously changing system state variable. This method ensures that the key for encrypting the data from the capacitive sensors is never static, significantly bolstering security against potential unauthorized access.

The process begins with the sensor attached to the urine bag, recording data such as volume output. This data is first encrypted using the generated encryption key specific to the patient. For instance, data from a sensor for ‘Patient123’ is encrypted with a key derived from their unique ID and the current system state, resulting in an encrypted format like 58d7af34bc. This encrypted data is then securely transmitted to our local server. The encryption is performed at the microcontroller level (ESP8266), not on an external computer. The microcontroller acquires raw sensor data, applies AES-256 encryption with dynamic key generation, and then transmits the encrypted data via Wi-Fi. The local server is primarily used for data storage, decryption, and access control management, rather than for initial encryption. Thus, the system is not dependent on a computer for real-time encrypted transmission. The local server setup plays a pivotal role in data handling and security. The server, running on a 12th Gen Intel(R) Core (TM) i7-12700K processor with 32.0 GB of RAM and a 1 TB SSD under Windows 11 Pro OS, is optimized for high-efficiency data processing and storage. The server is configured to serve as the central node for data reception, decryption, and storage and is fortified with network security measures, including firewalls and VPNs. Upon receiving the encrypted data, the server employs the reverse process for decryption. It dynamically generates the decryption key using the patient’s ID and the current system state, enabling it to decrypt the received data back to its original form. For example, the encrypted data 58d7af34bc. for ‘Patient123’ is decrypted to reveal the urine volume. Furthermore, the system integrates an OTP-based access control mechanism for data retrieval. Authorized medical personnel receive a time sensitive OTP linked to a patient’s ID for each access request, ensuring that data are encrypted and accessible only for a specific time frame.

The data is not only indicative but also stored for later analysis. Sensor readings are encrypted and transmitted to a local server, where they are stored in a structured database format (time-stamped CSV/SQL entries). This ensures that patient data can be retrieved and analysed for clinical decision-making and record-keeping. Raw sensor voltage values are stored, along with computed volume levels and alert logs. This dual storage approach allows both detailed technical review and simplified clinical interpretation. The mobile application primarily serves as a visualisation and alert interface. Data collection, transmission, encryption, and storage are autonomous processes, executed by the control unit and local server. The app does not need to be continuously active for monitoring or data storage; however, it must be running to receive real-time alerts or view live updates. The proposed system supports two modes: (i) Direct monitoring mode: The encrypted sensor data is sent from the microcontroller to the mobile app, which decrypts and displays it in real time, (ii) Storage and analysis mode: The encrypted data is also sent to a secure database on the server, allowing long-term storage and retrospective analysis. This dual-path architecture ensures both low-latency real-time monitoring (via the app) and secure archival of medical records (via the server database), which is essential in a healthcare context.

#### UO level monitoring algorithm

The overall working of the experimental setup is summarised in Algorithm 1. Sensors are initialized, and values are updated based on the sensor output voltages. The updated values can be viewed in the cell phone App anytime from any corner of the world. Concerned personnel are alerted if the sensor output values remain unchanged for about 6 h. This is because the minimum urination of a person is about 100 mL in 6 h. However, multiple alarms can be set based on the health conditions and monitoring requirements.

**Algorithm 1:**
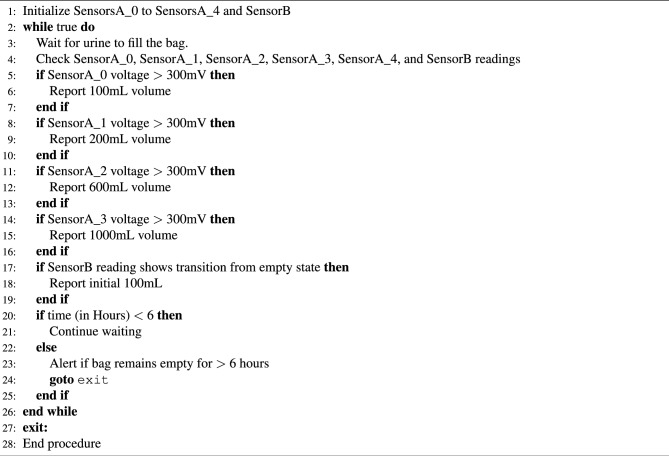
Initialize_sensors.

## Experimental results and discussions

This section presents the results obtained from the trials, where a series of experiments were conducted to validate the working of the developed system. The sensor outputs were carefully observed and documented through repetitive experiments conducted over a week to note any significant changes over time. However, there were no significant fluctuations in the recorded values. This test confirmed the accuracy of the proposed system.

**Fig. 6:**
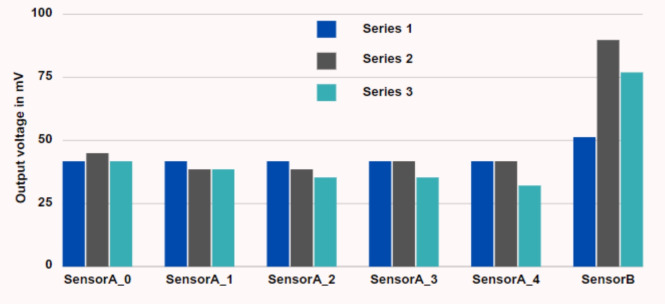
Sensor output when the bag is empty.

The experimental tests involved two scenarios: one involving a five-sensor electrode setup and another with a single-sensor electrode configuration, both fixed to the same urine bag. Each experiment continuously monitored sensors’ outputs over 10 min of precision. An initial measurement was recorded when the urine bag was empty and dry, representing its first use. Subsequently, the same measurements were replicated with the bag in an empty, wet condition (after saline water was emptied), to ascertain that moisture did not significantly impact the output voltage compared to the dry bag. Figure 6 presents a plot illustrating the average sensor output values in millivolts from three randomly selected trials, considering both the dry and wet states of the empty bag. Notably, in the case of the five-sensor electrode setup, no substantial variations were observed between the dry and wet conditions of the empty bag. Conversely, the single sensor electrode exhibited noticeable variations. The single electrode has a larger area causing a significant capacitance change due to change in dielectric between dry and wet conditions. These findings underscore the effectiveness of the five-sensor electrode architecture, particularly in the context of reusable urine bags.

**Fig. 7:**
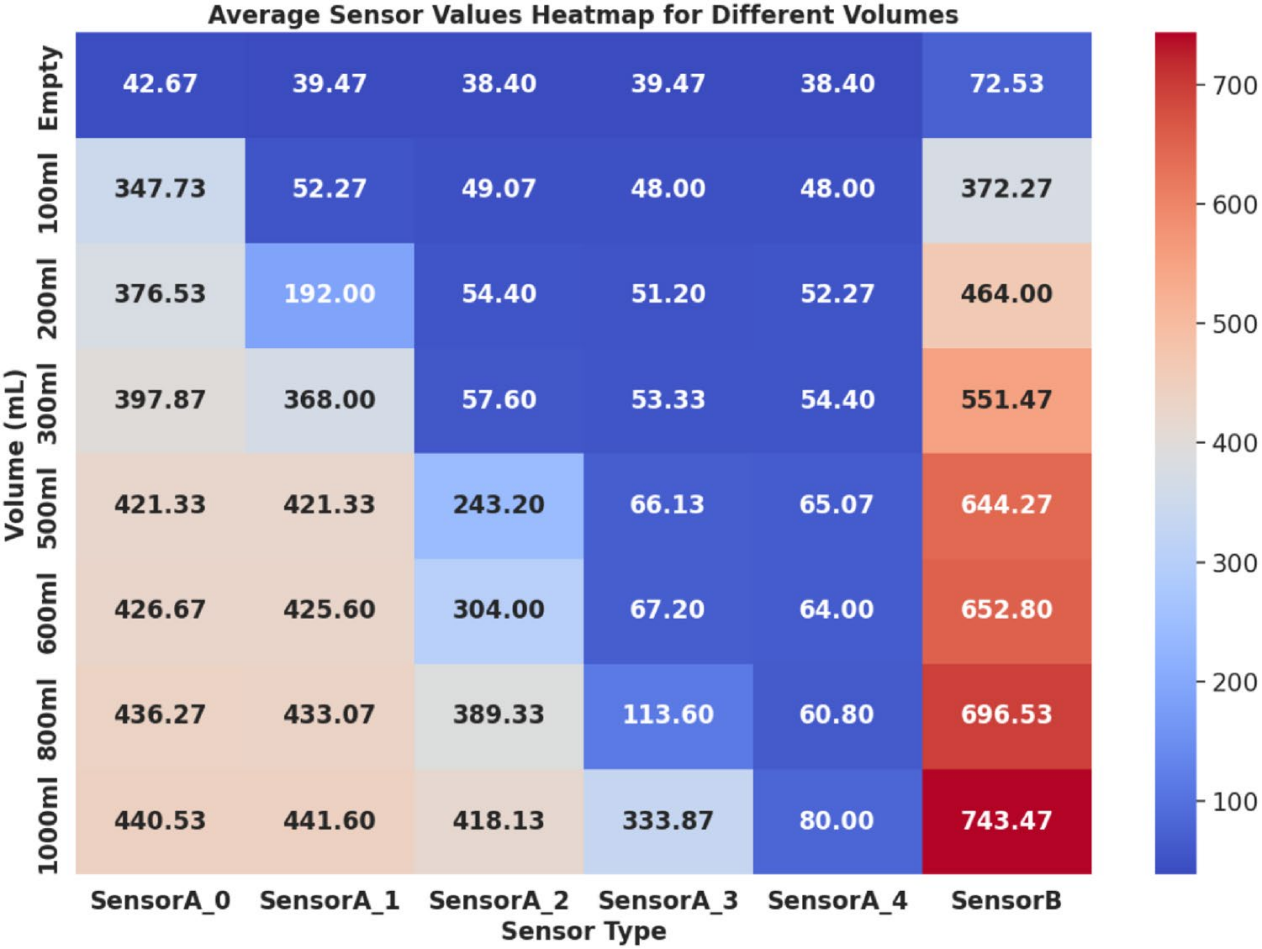
Sensor output for different volumes.

This study conducted experiments to assess sensor output variations in response to different urine volumes ranging from 100 to 1000 mL in increments of 100 mL. Figure 7 illustrates the sensor output values in millivolts specifically for 100 mL, 200 mL, 300 mL, 500 mL, 600 mL, 800 mL and 1000 mL. As the urine bag’s volume increases, corresponding changes are observed in the sensor output voltage levels. Notably, this increase in voltage is gradual and exhibits a non-linear relationship with volume growth. These significant voltage changes are attributed to the capacitive sensor configuration within the urine bag. The nonlinear behaviour is a consequence of the structural design of the urine bags, which leads to non-uniform changes in capacitance owing to varying distances between the sensor plates. It is worth noting that this nonlinearity is more pronounced in a single-electrode system than in a five-electrode system, where the sensor’s position provides a rough estimate of the volume level. Specifically, SensorA_0 exhibited a notable increase in output voltage when the volume reached 100 mL in the bag, followed by SensorA_1 at 200 mL, SensorA_2 at 600 mL, and finally, SensorA_3 exhibited an increase in output voltage at the 1000 mL volume level. However, the recorded values indicate variability rather than remaining constant. These minor fluctuations in the values are attributable to the movement of the bag and the shifting distances between the opposing surfaces of the bag. The bag was suspended and subjected to controlled movement to map to real-world usage conditions. Hence, this results in small fluctuations in the sensor output voltage values. However, volume estimation was approximated initially considering linear variations. For linear variations in volume, assuming the cylindrical structure of the bag, the volume per sensor is computed using the relation in Eq. ((6)).

6 $$\begin{aligned} \text {Volume per sensor} = \frac{\text {Bag capacity (in volume)}}{\text {Number of sensors}}. \end{aligned}$$

In the proposed system, five sensors are used, and the bag’s capacity is one litre. Hence, ideally, the sensors exhibit maximum voltage values (in mV) for an increase in every 200 mL. However, the volumetric distribution of the urine bag is nonlinear due to its structure. Notably, there are substantial and noteworthy disparities in the sensor output across SensorA_0 to SensorA_4 as the urine volume increases. This is due to the nonlinear bulging of the bag. SensorA_0 gets completely covered with 100 mL that is indicated by the output voltage value above 300 mV as shown in Fig. 7. Similarly, for 200 mL, a sudden change in the voltage value of sensor A_1 can be observed, and the value crosses 300 mV when the volume of the sample is greater or equal to 300 mL, which is a 200 mL rise from the sensor A_0s maximum. A similar trend was observed for the other sensors.

In contrast, such pronounced variations are not evident in the case of Sensor B. Specifically, Sensor B’s output voltage experiences a sharp transition from an empty state to 100 mL, but subsequent changes with increasing volume are less apparent. Consequently, it can be inferred that employing multiple sensors yields more accurate and reliable measurements of UO levels compared to relying solely on a single sensor.

In our analysis, we conducted statistical computations on the voltages obtained from the four lower sensors (SensorA_0 to SensorA_3) of the five sensor electrodes, as outlined in Table 2, to assess variations in sensor output voltage levels. We calculated the Mean, Skewness (Skw), and Standard Deviation (SD) for different volumes. When the bag is empty, the mean values for these sensors fall within the range of 38.4 to 42.67 mV, with standard deviations ranging from 1.85 to 3.4. These results indicate that all sensor values are consistent within this range when the bag is empty. Notably, as the volume increases, there is a rise in the mean values of these sensors.

**Table 2:** Sensor output voltage variations.

Quantity	Sensor A_0			Sensor A_1			Sensor A_2			Sensor A_3		
	Mean	Skw	SD (mV)	Mean	Skw	SD (mV)	Mean	Skw	SD (mV)	Mean	Skw	SD (mV)
Empty	42.67	0.7	1.85	39.47	0.7	1.85	38.4	0.004	3.0	39.47	-0.7	3.4
100 mL	347.7	−0.5	24.85	52.27	−0.5	6.67	49.07	0.7	1.84	48	–	0
200 mL	376.5	−0.7	7.3	192	−0.7	16.62	54.4	–	0	51.2	−1	8.7e−15
600 mL	426.7	0.38	4.8	425.6	0.6	8.4	304	−0.33	50.29	67.2	0.6	8.4
1000 mL	440.5	−0.24	8.05	441.6	0.7	11.1	418.1	−0.24	8.05	333.9	0.69	65.76

**Fig. 8:**
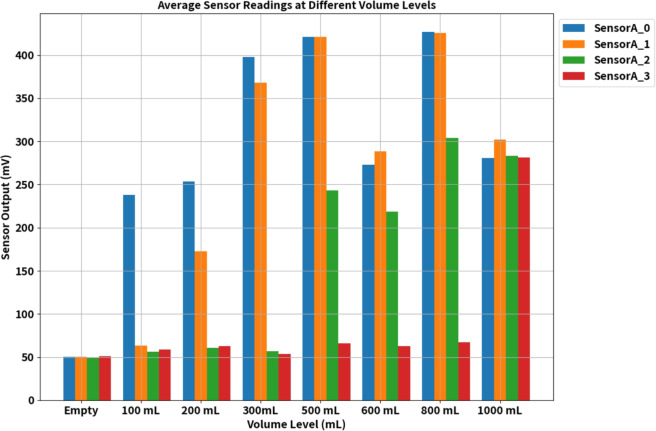
Average values of the sensors at different volume.

As mentioned earlier, we conducted experiments at various volume levels, and Fig. 8 illustrates the average voltage values obtained from all four sensors. There is a gradual increment in sensor output voltages as the volume progressively increases. Specifically, SensorA_0 displayed a range of voltage variation from 50.49 to 280.61 mV as the volume increased from 100 to 1000 mL. In contrast, significant voltage fluctuations in SensorA_3 were only noticeable at the 1000 mL volume level.

**Fig. 9:**
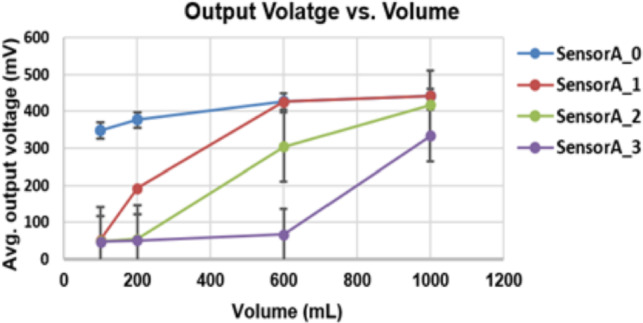
Correlation between sensor average output voltage and urine bag volume (100–1000 mL) representing measurement uncertainty.

The relationship between urine bag volume (100–1000 mL) and the average output voltage of the capacitive sensors with error bars representing the standard deviation across repeated trials is presented in Fig. 9. The results demonstrate a clear correlation between sensor voltage and fluid volume, although the relationship is nonlinear due to bag deformation and dielectric effects, as discussed earlier. Sensor$A_0$ shows a pronounced response at 100 mL, while higher-level sensors ($A_1$ to $A_3$) exhibit distinct voltage changes at larger volumes. The error bars reflect uncertainties arising from bag movement, measurement noise, and small capacitance fluctuations, yet remain within acceptable limits for clinical monitoring. Importantly, the strong general correlation between estimated and actual volumes (PCC = 0.9986) confirms the reliability of the system. These findings reinforce the potential of the proposed approach to provide accurate and repeatable urine output measurements under real-world conditions.

**Fig. 10:**
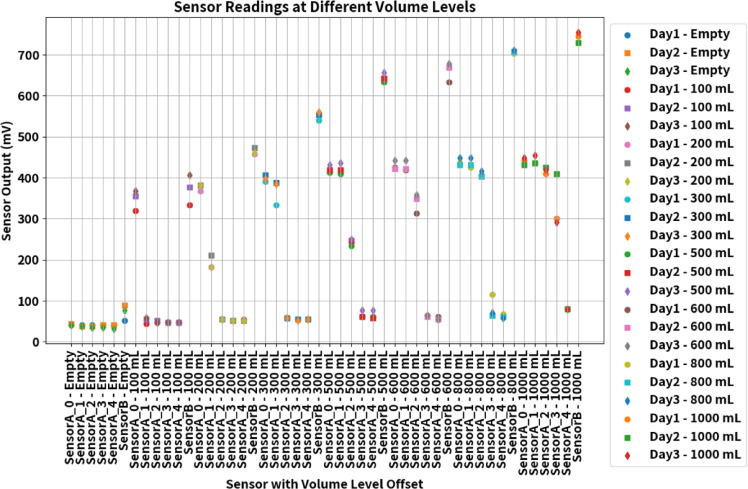
Average variations of sensor voltage at different volumes recorded on different days.

The study also focused on monitoring sensor output voltages at different volume levels through repeated experiments conducted over three days. The data acquisition process involved recording ADC values for approximately 150 s for each experiment and calculating average values for each sensor at various volume levels. The analysis, as depicted in Fig. 10, demonstrates that the variation in the offset voltage is negligible, confirming the stability of the sensor output over the testing period. This reflects the reliability of the sensors under varying conditions, which is of its practical applications.

**Fig. 11:**
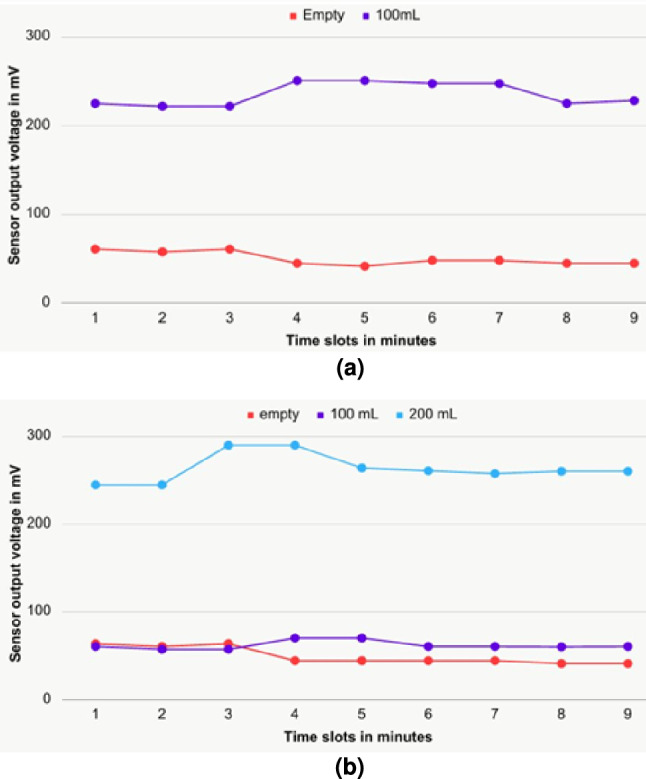
Fluctuation in sensor output voltages with time slots; (**a**) sensorA_0, (**b**) sensorA_1.

Output voltage levels of the sensors are subjected to variations due to several factors, such as the movement of the bag, temperature variations, etc. Hence, it is crucial to note the significant value variations for planning a calibration method. However, the variations are not significant, as shown in Fig. 10. To illustrate the variations in sensor output voltages, a graphical representation of the voltage variations recorded as time series data is provided in Fig. 11, and it provides an expanded view of Fig. 10, for only two sensors: sensorA_0 and sensorA_1. However, all the sensors exhibited small variations during the time slots, as depicted in Fig. 10. The plot is shown for 9 min because the output voltage remained almost constant after this duration. It is evident from Fig. 11 that the variations are minimal; hence, a calibration circuit is not necessary to estimate the UO levels. These plots further confirm the reliability of the sensors in practical scenarios.

We conducted a series of experiments using water to validate the accuracy of the capacitive measurement method, which relies on factors such as distance and dielectric constant. Given the disparity in dielectric constants between water and saline water, we anticipated differences in sensor voltage levels when measuring water. Table 3 data represents the variations in sensor output voltage for water. By comparing the values in Tables 2 and 3, it becomes evident that there is a distinct contrast in the output voltage values between water and saline water. Consequently, the proposed capacitive sensor-based automated and remote urine output level monitoring system holds promise as a sustainable solution for enhancing patient monitoring workflows in both ICU and home care settings.

**Table 3:** Output voltage variations of sensors for water.

Quantity	Sensor 0			Sensor 1			Sensor 2			Sensor 3		
	Mean	Skw	SD (mV)	Mean	Skw	SD (mV)	Mean	Skw	SD (mV)	Mean	Skw	SD (mV)
Empty	50.49	0.55	7.56	50.49	0.67	9.80	49.05	0.49	9.77	50.85	0.85	9.50
100 mL	238.5	−0.28	14.17	63.1	0.72	5.54	56.23	0.95	3.14	58.47	0.27	2.89
200 mL	253.7	−0.29	16.29	172.6	−0.04	17.44	60.7	−2.04	1.20	62.61	0.29	1.77
600 mL	272.9	−0.2	26.87	288.6	−0.3	31.17	218.8	−0.94	21.22	62.66	0.12	9.68
1000 mL	280.7	0.4	30.96	302.3	0.4	33.86	283.6	0.41	40.25	281.3	0.37	44.52

Finally, Pearson Correlation Coefficient (PCC) is calculated to assess the urine bag’s estimated volume as given in Eq. ((7)). We obtained a PCC of 0.9986, indicating a strong correlation between the estimated volume and actual volume in the urine bag.

7 $$\begin{aligned} {\rm PCC} = \sum \frac{({\rm AV}-M_{\rm EV})({\rm EV}-M_{\rm AV})}{\sqrt({\rm DS}_{\rm AV})({\rm DS}_{\rm EV})}, \end{aligned}$$

where AV is the actual volume of the saline measured using a measuring jar, EV is the estimated volume during the experimentation, MAV and MEV are mean values, and DSAV and DSEV are deviation squared values of the actual and estimated volume.

**Fig. 12:**
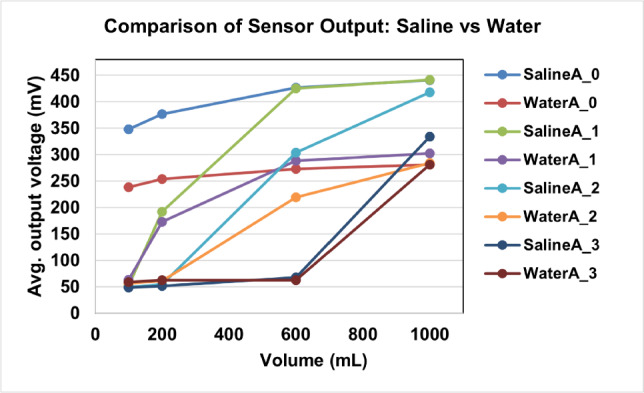
Overlay of saline vs. water results for sensors $A_0 - A_3$.

The comparison of the sensor output voltages for saline and water in 100–1000 mL volumes is illustrated in Fig.12. The results show that saline consistently produces higher voltage readings than water for the same volume, which can be attributed to its higher dielectric constant ($\epsilon = 78.4$ for saline vs. $\epsilon = 80$ for water, but modified by ionic conductivity effects). This confirms that the capacitive sensing method is sensitive to fluid dielectric properties in addition to volume. Although both fluids demonstrate a clear correlation between sensor output and volume, the distinction between saline and water responses highlights the importance of calibration when deploying the system in clinical contexts. Importantly, relative trends between sensors ($A_0-A_3$) remain consistent between fluids, strengthening the robustness of the sensing approach. These results demonstrate that the system is capable of differentiating between fluids and reliably tracking urine volume, with calibration strategies that offer a pathway to further improve accuracy.

The proposed non-invasive method for remote UO monitoring represents a viable solution for enhancing patient monitoring systems. Notably, the key advantage is its compatibility with existing market-available urine bags, eliminating the need for custom manufacturing. Furthermore, this solution offers a compact and cost-effective design. Within this proposed system, there is flexibility in setting up alarm triggers tailored to individual patient conditions, and the bags can be reused after emptying. Importantly, this non-invasive method ensures no direct contact between the measurement system and the patient’s body. The incorporation of wireless technology and a mobile application enables the simultaneous monitoring of multiple patients from any location, eliminating the requirement for an additional workforce. While similar proposals for automated UO monitoring devices exist in the literature, they often rely on Bluetooth or wired communication technology^3,24,26^, limiting their range and obstructing remote monitoring capabilities.

In contrast, the wireless technology utilized in the proposed approach allows for remote UO monitoring. Additionally, the mobile application enables regular monitoring of patients in both hospital and home settings. A previous study^3^ employed capacitive sensors for automated UO monitoring but utilized Bluetooth and commercially available sensors. Our approach used cost-effective thin copper tapes to develop a more affordable device. Consequently, leveraging wireless technology, our proposed patient monitoring system empowers healthcare professionals to routinely monitor patients’ UO levels, facilitating more timely data collection, reducing human errors, and alleviating the documentation workload for caregivers. Unlike previous studies that required customized containers or bags, the proposed system works with standard urine bags available in the market. This feature significantly enhances the proposed system’s practicality and ease of adoption. Remote monitoring capabilities of the proposed system enable remote UO monitoring, a feature absent in many systems listed in comparison Table 4. Additionally, a smartphone application for monitoring, coupled with a Wi-Fi-enabled control unit, offers a significant advancement over other systems with limited range due to Bluetooth connectivity or lack of wireless transmission capabilities. The proposed system is notably cost-effective, with an estimated cost of 15 USD. Another salient feature of the proposed system is AES-256 encryption and dynamic key generation for secure data transmission, addressing data privacy and security.

**Table 4:** Comparison of the proposed UO level monitoring with earlier studies.

Author year	Type of sensor used and framework	Estimated cost	Mode of communication	Salient features
Otero et al.^3^	A capacitive sensor; measurement of the UO collected within a rigid urine container.	–	Bluetooth	Invasive; need for customised container; no remote monitoring; limited by the distance; not addressed data security
Otero et al.^24^	Flow sensor; sensing and supervising two urine containers of different volumes.	–	Bluetooth	Non-Invasive; need for customised containers; no remote monitoring; limited by the distance; not addressed data security
Lee et al.^25^	Weight sensor; device measures UO in 1-sec interval range with a customised urine bag.	26 USD	Wi-Fi enabled microcontroller	Non-Invasive; need for customised urine bag; no remote monitoring; not limited by the distance; not addressed data security
Otero et al.^26^	Float sensor; monitors filling of two urine containers of 15 ml and 180 ml sizes.	351 USD	Bluetooth	Non-Invasive; need for customised urine container; no remote monitoring; limited by the distance; not addressed data security
Stephannie et al.^27^	Coaxial capacitive sensor; only level measurements using the sensor.	–	Wired connection	Invasive; no data transmission
**Proposed system**	Capacitive sensors mounted on existing urine bags; continuously monitors UO level.	15 USD	Wireless with a dedicated mobile app	Non-Invasive; no need for customised urine bag/container; remote monitoring; not limited by the distance; addressed data security

It is important to acknowledge that the present validation is carried out using water and saline as controlled test fluids. While these fluids are valuable for establishing baseline performance and isolating dielectric effects under repeatable laboratory conditions, they do not fully represent the complex and variable dielectric properties of actual urine. Real urine exhibits variations in solute concentration, pH, and ionic composition, all of which may influence sensor response. Therefore, testing with actual urine samples is essential for full clinical validation. As part of future work, we plan to conduct experiments with human urine under appropriate ethical approval, enabling calibration strategies that account for patient-specific variability. Such studies will further substantiate the clinical applicability of the proposed system and may open avenues for extending its functionality toward monitoring additional urine parameters.

## Conclusion

This work presents a secure, cost-effective, and non-invasive urine output monitoring system designed to improve patient management in both hospital and home care contexts. The system employs copper tape-based capacitive sensors mounted on standard urine bags, with outputs validated across 100–1000 mL volumes. Experimental results confirmed strong agreement between estimated and actual volumes (PCC =0.9986), with stable performance under repeated trials and both wet and dry conditions. With an estimated cost of only  15 USD, the proposed solution is significantly more affordable than comparable systems. In addition to functional accuracy and low cost, the system incorporates advanced security features, including AES-256 encryption with dynamic key generation and OTP-based access control, ensuring secure and compliant transmission of patient data. The Wi-Fi-enabled control unit and dedicated mobile application provide real-time monitoring and timely alerts, reducing the workload of healthcare staff while improving patient safety. The future work will focus on conducting large-scale clinical trials to evaluate system performance in real patient environments, integrating the device with hospital electronic health record (EHR) systems for seamless data management, miniaturising the hardware for more compact and portable use, and extending the sensing capabilities to monitor additional urine parameters, such as pH or specific biomarkers, for enhanced diagnostic value.

## Data Availability

The datasets used and/or analyzed during the current study are available from the corresponding author on reasonable request.

## References

[1] Abate, S. M., Ahmed, Ali S., Mantfardo, B. & Basu, B. Rate of Intensive Care Unit admission and outcomes among patients with coronavirus: A systematic review and Meta-analysis. *PLoS ONE* **15**(7), e0235653. 10.1371/journal.pone.0235653 (2020). 32649661 10.1371/journal.pone.0235653 PMC7351172

[2] Poncette, A. S. et al. Improvements in patient monitoring in the intensive care unit: Survey study. *J. Med. Internet Res.* **22**(6), e19091. 10.2196/19091 (2020). 32459655 10.2196/19091 PMC7307326

[3] Otero, Abraham, Apalkov, Andrey, Fernández, Roemi & Armada, Manuel. A new device to automate the monitoring of critical patients’ urine output. *Biomed. Res. Int.* **2014**, 587593. 10.1155/2014/587593 (2014). 24605331 10.1155/2014/587593 PMC3925530

[4] Akben, S. B. Early stage of chronic kidney disease by using statistical evaluation of the previous measurement results. *Biocybern. Biomed. Eng.* **36**(4), 626–631. 10.1016/J.BBE.2016.08.004 (2016).

[5] ...Kruk, M. E. et al. High-quality health systems in the sustainable development Goals era: Time for a revolution. *Lancet Glob. Health* **6**(11), e1196–e1252. 10.1016/S2214-109X(18)30386-3 (2018). 30196093 10.1016/S2214-109X(18)30386-3 PMC7734391

[6] Munagala, N. K., Langoju, L. R. R., Rani, A. & Reddy, D. V. R. K. A smart IoT-enabled heart disease monitoring system using meta-heuristic-based Fuzzy-LSTM model. *Biocybern. Biomed. Eng.* **42**(4), 1183–1204. 10.1016/j.bbe.2022.10.001 (2022).

[7] Julakanti Varsha, I. V., Tarun Raj, S. S. & Krishna Chaithanya, J. Design of automated health monitoring system using IoT. *ECS Trans.* **107**(1), 13131. 10.1149/10701.13131ecst (2022).

[8] Bhardwaj, V., Joshi, R. & Gaur, A. M. IoT-based smart health monitoring system for COVID-19. *SN Comput. Sci.* **3**(2), 137. 10.1007/s42979-022-01015-1 (2022). 35079705 10.1007/s42979-022-01015-1 PMC8772261

[9] Vedaei, S. S. et al. COVID-SAFE: An IoT-based system for automated health monitoring and surveillance in post-pandemic life. *IEEE Access* **8**, 188538–188551. 10.1109/ACCESS.2020.3030194 (2020). 34812362 10.1109/ACCESS.2020.3030194 PMC8545279

[10] Khan, M. M. et al. IoT-based smart health monitoring system for COVID-19 patients. *Comput. Math. Methods Med.* **2021**, 8591036. 10.1155/2021/8591036 (2021). 34824600 10.1155/2021/8591036 PMC8610655

[11] Narendra Swaroop, K., Chandu, K., Gorrepotu, R. & Deb, S. A health monitoring system for vital signs using IoT. *Internet of Things* **5**, 116–129. 10.1016/j.iot.2019.01.004 (2019).

[12] Maurya, L., Maurya, L., Mahapatra, P. K., Mahapatra, P. K. & Chawla, D. Non-contact breathing monitoring by integrating RGB and thermal imaging via RGB-thermal image registration. *Biocybern. Biomed. Eng.* **41**(3), 1107–1122. 10.1016/J.BBE.2021.07.002 (2021).

[13] Das, P., Deka, Ra. ., Sengyung, S., Nath, B. K. R. & Bordoloi, H. A review paper on patient monitoring system. *J. Appl. Fundam. Sci.* **1**, 264–267 (2015).

[14] Shubhangi, M. Real time health monitoring using GPRS technology. *Int. J. Comput. Sci. Netw. (IJCSN)* **1**, 1–8 (2012).

[15] Baby Shalini, V. Smart health care monitoring system based on Internet of Things (IoT). In *International Conference on Artificial Intelligence and Smart Systems (ICAIS-2021)*, pp. 1449-1453 (2021).

[16] Mrozek, Dariusz, Koczur, Anna & Małysiak-Mrozek, Bo. żena. Fall detection in older adults with mobile IoT devices and machine learning in the cloud and on edge. *Inf. Sci.* **537**, 132–147 (2020).

[17] Minor, J., Smith, A., Deutsch, F. & Kellum, J. A. Automated versus manual urine output monitoring in the intensive care unit. *Sci. Rep.* **11**, 17429. 10.1038/s41598-021-97026-8 (2021). 34465821 10.1038/s41598-021-97026-8 PMC8408210

[18] Willner, D. et al. Early identification of acute kidney injury in the ICU with real-time urine output monitoring: A clinical investigation. *BMC Nephrol.* **22**(1), 293. 10.1186/s12882-021-02485-w (2021). 34445954 10.1186/s12882-021-02485-w PMC8394570

[19] Otero, A., Palacios, F., Akinfiev, T. & Apalkov, A. A low-cost device for monitoring the urine output of critical care patients. *Sensors (Basel)* **10**(12), 10714–32. 10.3390/s101210714 (2010). 22163495 10.3390/s101210714 PMC3231093

[20] Kushnir, Alexander et al. Improving fluid output monitoring in the intensive care unit. *J. Intensive Care Med.* **37**(1), 114–119. 10.1177/0885066620979663 (2022). 33292043 10.1177/0885066620979663

[21] Hao, W., Hao, X. & Yang, C. Design and optimization of urinary real-time nursing model based on medical internet of things. *Comput. Intell. Neurosci.* **20**(2022), 7067856. 10.1155/2022/7067856 (2022). 10.1155/2022/7067856 PMC9045976 35498189

[22] Munish, B., Simranpreet, K. & Sandeep, K. S. IoT-inspired smart toilet system for home-based urine infection prediction. *ACM Trans. Comput. Healthc.* **1**(3), 1–25. 10.1145/3379506 (2020).

[23] Tasoglu, S. Toilet-based continuous health monitoring using urine. *Nat. Rev. Urol.* **19**, 219–230. 10.1038/s41585-021-00558-x (2022). 35064251 10.1038/s41585-021-00558-x

[24] Otero, A., Fernández, R., Apalkov, A. & Armada, M. An automatic critical care urine meter. *Sensors* **12**(10), 13109–13125. 10.3390/S121013109 (2012). 23201988 10.3390/s121013109 PMC3545559

[25] Yeh, H.-J.J. An IoT-based automatic and continuous urine measurement system. *BioMedInformatics* **3**(2), 446–454. 10.3390/biomedinformatics3020030 (2023).

[26] Otero, A., Panigrahi, B., Palacios, F., Akinfiev, T. & Fernandez, R. A prototype device to measure and supervise urine output of critical patients. *Intechopen* **321–334**, 2009 (2009).

[27] Mathews, S. C., Thattai, K., Ramanathan, P. K. & Marimuthu, R. Design and development of a simple and efficient low-cost embedded liquid level measurement system. *Int. J. Eng. Technol. (IJET)* **5**(2), 734–741 (2013).

